# Durrer prizes 2011

**DOI:** 10.1007/s12471-012-0288-4

**Published:** 2012-05-11

**Authors:** E. E. van der Wall, M. J. Schalij

**Affiliations:** Department of Cardiology, Leiden University Medical Center, PO Box 9600, Leiden, the Netherlands

For the sixth time in a row, the Netherlands Heart Journal (NHJ), the official journal of the Netherlands Society of Cardiology (NVVC), awarded the Durrer Prize to the best two articles published in the year 2011.

In 2006, it was thought appropriate to set up a special publication prize to stimulate the submission of original articles to NHJ. Two articles per year are selected, one more basically oriented, and one with a mainly clinical focus.

The publication prize carries the name of one of the fathers of Dutch Cardiology, Prof. Dr. Dirk Durrer (1918–1984, Head of the Department of Cardiology in the ‘Wilhelmina Gasthuis’, Amsterdam), who performed pioneering work in the field of electrical activation of the heart in the 1960s.

From a total of more than 30 original articles published in NHJ in 2011, we selected as best basic article: Invasive coronary imaging in animal models of atherosclerosis, by Van Ditzhuijzen et al. from Erasmus University Medical Center Rotterdam and ICIN, Utrecht [1].

As best clinical article we selected: Percutaneous renal denervation for the treatment of resistant essential hypertension: the first Dutch experience, by Voskuil et al. from University Medical Center Utrecht [2].

At the annual spring meeting of the NVVC, held in Noordwijkerhout on 12 April, the first authors of both articles received a nice little statue of an owl (Prof. Durrer’s favourite animal) and an educational grant provided by the NVVC. We would like to congratulate the authors with their awards, and we thank them for sending their excellent work to NHJ.
